# Antibacterial activity of native California medicinal plant extracts isolated from *Rhamnus californica* and *Umbellularia californica*

**DOI:** 10.1186/s12941-015-0086-0

**Published:** 2015-05-23

**Authors:** Maria G Carranza, Mary B Sevigny, Debashree Banerjee, Lacie Fox-Cubley

**Affiliations:** Department of Natural Sciences and Mathematics, Dominican University of California, 50 Acacia Avenue, San Rafael, CA 94901 USA

**Keywords:** Methicillin-resistant S. aureus, Medicinal plants, Antimicrobial activity, Rhamnus californica, Umbellularia californica

## Abstract

**Background:**

Antimicrobial resistance (AMR) is a major threat to global public health. Medicinal plants have long been used as remedies for infectious diseases by native cultures around the world and have the potential for providing effective treatments for antibiotic-resistant infections. *Rhamnus californica* (Rhamnaceae) and *Umbellularia californica* (Lauraceae) are two indigenous California plant species historically used by Native Americans to treat skin, respiratory and gastrointestinal infections. This study aimed to assess the *in vitro* antimicrobial activity of methanolic extracts of leaves and bark of *R.* and *U. californica* against methicillin-resistant *Staphylococcus aureus* (MRSA) and other Gram-positive and Gram-negative bacteria.

**Methods:**

Methanolic extracts of leaves and bark of *R.* and *U. californica* were prepared by soxhlet extraction and evaluated for their antimicrobial activity against *Bacillus cereus*, *Streptococcus pyogenes*, *Mycobacterium smegmatis*, *Staphylococcus aureus*, MRSA, *Escherichia coli* and *Pseudomonas aeruginosa* using disc diffusion and minimal inhibitory concentration (MIC) assays. Chemical profiling of the extracts was performed using standard methods.

**Results:**

All extracts inhibited the growth of MRSA and other Gram-positive bacteria with MICs of 3.3-6.0 mg/ml. Gram-negative organisms were unaffected by these extracts. *U. californica* extracts (leaves and bark) had the lowest MIC values. Chemical profiling detected the presence of quinones, alkaloids, flavonoids, cardenolides, tannins and saponins in these extracts. Our study is the first to report the antimicrobial properties of *R.* and *U. californica* and illustrates their promising anti-MRSA potential.

**Conclusions:**

Our results give scientific credence to the traditional medicinal uses of these plants by the indigenous peoples of California. Further investigation of the secondary metabolites responsible for the antimicrobial activity of these extracts against MRSA is warranted.

## Background

The incidence of infections by antibiotic-resistant bacteria has increased significantly over the last decade. In the U.S., over 90,000 infections per year are caused by methicillin-resistant *Staphylococcus aureus* (MRSA) alone [1]. Antimicrobial resistance (AMR) has also been found in strains of *Streptococcus pneumonia*, *Escherichia coli*, *Pseudomonas aeruginosa*, and *Mycobacterium tuberculosis*, to name a few. The emergence of AMR has prompted the Centers for Disease Control (CDC) and the World Health Organization (WHO) to spearhead efforts aimed at combating this dilemma through public education about the misuse of antibiotics, increased surveillance, and research focused on developing new, more effective therapies [2]. For thousands of years native peoples across the globe have used traditional medicine to treat many human diseases, including infectious diseases [3]. Native Americans produced traditional medicines from approximately 2700 species of vascular plants [4]. The medicinal ethnobotanical knowledge of Native Americans has been an important source for the identification of bioactive plants, and studies show a high degree of correlation between traditional medicinal uses and observed biological activity [5].

**Fig. 1:**
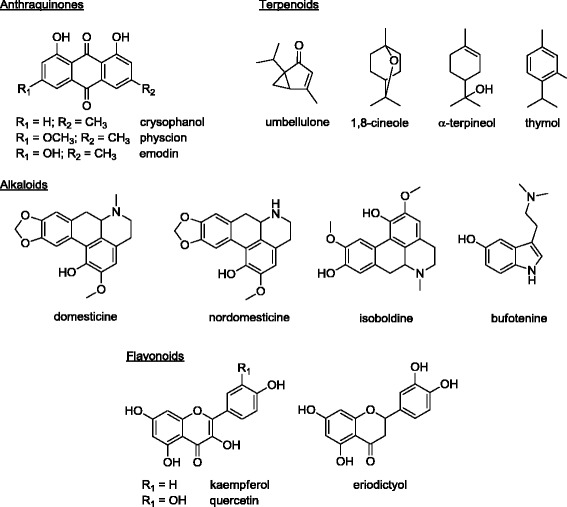
Selected examples of reported structural classes from *R. californica* and *U. californica*

*Rhamnus californica* (Rhamnaceae) and *Umbellularia californica* (Lauraceae) are two indigenous California plant species historically used by Native Americans for their medicinal properties [6, 7]. Specifically, *R. californica* was used as a decoction of leaves for the treatment of poison oak dermatitis (Costanoan tribe); crushed leaves and berries were used to heal infected sores, burns and wounds (Kawaiisu tribe); and a decoction of bark was used to treat grippe (Mendocino and Yokia tribes) [4, 8]. Similarly, *U. californica* was used as a decoction of leaves and as a steam bath for the treatment of cold symptoms (Karok, Pomo and Kashaya tribes); a poultice of leaves was applied to sores (Karok, Pomo and Kashaya tribes) and used as a treatment for poison oak dermatitis (Costanoan tribe) [9]; a decoction of leaves was used for sore throat and chest congestion (Pomo, Kashaya tribes); crushed leaves were used for nasal decongestion (Yuki tribe); and a decoction of whole plant was taken for stomachaches (Mendocino tribe) [4, 8]. Earlier chemical studies reported the presence of anthraquinones [10, 11] in the genus *Rhamnus*, while terpenoids [9, 12], alkaloids [13], and flavonoids [14] are reported as major constituents of *U. californica* [15] with cyanogens, sugars and tannins as minor constituents [12]. Selected examples of reported structural classes from *R.* and *U. californica* are shown in Fig. 1.

The use of both species in Native American traditional medicine for the treatment of symptoms associated with skin, respiratory and gastrointestinal infections provided a rationale for investigating the *in vitro* antimicrobial potential of the methanolic extracts of leaves and bark of *Rhamnus californica* (Coffeeberry) and *Umbellularia californica* (California bay or California laurel) against MRSA and other types of Gram-positive and Gram-negative bacteria. We are the first to report the antibacterial activity of extracts from these two species and illustrate their promising anti-MRSA potential.

## Methods

### Plant material and extraction

*Rhamnus californica* and *Umbellularia californica* were obtained from the University of California at Berkeley Botanical Garden (UCBBG). Specimens were authenticated by Dr. Holly Forbes, curator of the UCBBG. Voucher specimens for each species (50.1622 and 71.0162) have been deposited at the UC Berkeley Herbarium. Leaves and bark were separated and oven dried after which they were grinded into fine powder or shavings. Thimbles were filled with dried leaf or bark material (30 g) and extracted using a Soxhlet apparatus. The plant materials were successively extracted with hexanes, dichloromethane, and methanol (2 × 100 ml for 24 h each). The resulting extracts were filtered, concentrated under reduced pressure, and kept at 4 °C.

### Microorganisms tested

All bacterial organisms were purchased from the American Type Culture Collection (ATCC; Manassas, VA, USA). Specific strains used were: *Bacillus cereus* ATCC® 14579™, *Escherichia coli* ATCC® 25922™, *Mycobacterium smegmatis* ATCC® 10143™, *Pseudomonas aeruginosa* ATCC® 10145™, *Staphylococcus aureus* ATCC® 25923™, methicillin-resistant *Staphylococcus aureus* (MRSA) ATCC® BAA-1683™, and *Streptococcus pyogenes* ATCC® 10782™.

### Disc-diffusion assay

The disc diffusion method developed by Kirby and Bauer was used with some modifications [16]. Initially, bacterial cultures were streaked onto tryptic soy agar plates and incubated overnight at 37 °C to obtain individual colonies. Colonies were then selected and resuspended in tryptic soy broth to achieve turbidity equal to that of a 0.5 McFarland Turbidity standard (BD Biosciences, Franklin Lakes, NJ, USA). Using a cotton swab, the bacterial suspension was used to inoculate a Mueller-Hinton agar plate (Carolina Biological Supply Company, Burlington, NC, USA). Various doses of plant extracts (all dissolved in methanol) were applied to 6 mm diameter paper discs (BD Biosciences), and the discs were then carefully placed onto the surface of the Mueller-Hinton agar plate. Plates were incubated at 35 °C for 20–22 h, after which the diameters of the zones of inhibition around each disc were measured. Streptomycin discs (10 μg; BD Biosciences) were used as a positive control.

### Minimal Inhibitory Concentration (MIC) assay

The microplate method developed by J.N. Eloff was used, with some modifications, to test the MIC of each extract for each bacterial species that demonstrated susceptibility in the Kirby-Bauer assay [17]. Briefly, bacterial colonies growing on either tryptic soy or brain heart infusion (BHI) agar plates were selected and resuspended in Mueller-Hinton broth (Carolina Biological Supply Company) to achieve turbidity equal to that of a 0.5 McFarland Turbidity standard. In a 96-well plate, 50 μl aliquots of this bacterial suspension were mixed with various concentrations of each extract (or methanol, as a negative control) for a final volume of 100 μl per well. The plate was incubated at 35 °C for 20–22 h. Due to the slow growth of *M. smegmatis*, that particular assay plate was incubated for 46–48 h. In order to better visualize bacterial growth, 40 μl of 0.2 mg/ml *p*-iodonitrotetrazolium violet (INT; Sigma-Aldrich Corp., St. Louis, MO, USA) were then added to each well, and the plate was incubated at 37 °C for another 30 min. Wells were analyzed visually for the development of a reddish hue, which would indicate bacterial growth.

### Chemical profiling

Qualitative detection of alkaloids, flavonoids, leucoantocyanidins, cardenolides, tannins, saponins, steroids and quinones was performed using standard procedures [18–21]. Each assay was performed in triplicate.

## Results

### Antimicrobial activity

The antimicrobial activity of the three methanolic extracts was initially assessed using the Kirby-Bauer disc diffusion assay. Extract doses ranging from 1 to 25 mg were tested on the Gram-negative organisms *E. coli* and *P. aeruginosa* and on the Gram-positive bacteria *B. cereus*, *S. pyogenes*, *S. aureus*, and MRSA. Streptomycin (10 μg/disc) was used as a positive control, and 100 % methanol, the extract solvent, was used as negative control. The Gram-negative bacteria were virtually unaffected by the extracts. However, all of the Gram-positive bacteria demonstrated sensitivity to each extract at doses between 1 and 5 mg (Table 1). Of the three extracts, the one from *R. californica* leaves appeared to be the most active. Interestingly, its greatest effect was seen on the only antibiotic-resistant strain tested— MRSA.

**Table 1:** Antimicrobial activity of methanolic extracts of R. californica and U. californica analyzed by Kirby-Bauer disc-diffusion assay

Bacterial Strains							
Diameter of Zone of Inhibition (mm)							
Plants	Dose (mg)	*B. cereus* ATCC® 14579	*E. coli* ATCC® 25922	*P. aeruginosa* ATCC® 10145	*S. aureus* ATCC® 25923	*MRSA* ^1^ ATCC® BAA-1683	*S. pyogenes* ^1^ ATCC® 10782
*R. californica*							
Leaves	1	10.0 ± 2.1	--	--	--	10.0	9.0
	5	12.3 ± 0.5	--	--	11.0 ± 1.2**	15.0	12.0
	10	13.0 ± 0.0	--	--	12.5 ± 2.1^Ψ^	16.0	14.0
	25	14.3 ± 0.5	--	--	14.0 ± 1.4**	n.t.	n.t.
*U. californica*							
Leaves	1	9.0 ± 0.0	--	--	--	8.0	8.0
	5	9.5 ± 0.5	--	--	9.3 ± 1.0**	9.0	11.0
	10	9.3 ± 0.5	--	--	10.5 ± 1.4^Ψ^	10.0	15.0
	25	10.0 ± 0.0	--	--	10.9 ± 2.0**	n.t.	n.t.
Bark	1	--	--	--	8.0 ± 0.0^Ψ^	--	--
	5	9.3 ± 0.5	--	--	12.8 ± 2.6**	9.0	8.0
	10	10.3 ± 0.5	--	--	17.5 ± 1.4^Ψ^	10.0	11.0
	25	11.7 ± 0.5	--	--	16.0 ± 1.2**	n.t.	n.t.
Streptomycin	0.01	23.8 ± 1.2	19.8 ± 0.2	10.4 ± 0.3	18.9 ± 1.0	17.0 ± 0.0	18.0 ± 0.0
Methanol	100 %	--	--	--	--	--	--

Data represented as averages ± SD. --, no significant zone of inhibition; n.t., not tested; ^1^, *n* = 1; Ψ, *n* = 2; **, *n* = 4; remaining data (*B. cereus*, *E. coli*, and *P. aeruginosa*) represent sample size of 3 (*n* = 3)

### Minimal Inhibitory Concentration (MIC)

After having determined that all the extracts significantly inhibited the growth of MRSA and other Gram-positive organisms, minimal inhibitory concentration (MIC) assays were performed (Table 2). Due to difficulty culturing *S. pyogenes* within the 96-well plate, the MIC assay could not be carried out on this organism. For the remaining four organisms, MIC values ranged between 3.3 and 6.0 mg/ml. *B. cereus* exhibited the greatest sensitivity to the three extracts, and MRSA predictably required slightly higher concentrations to inhibit growth. Although difficulty in growing a lawn of *M. smegmatis* prevented it from being tested using the Kirby-Bauer assay, the MIC assays demonstrated that even this acid-fast organism was susceptible to the extracts. Additionally, according to these MIC data, the *U. californica* extracts, rather than the *R. californica* extract, appeared to be most active.

**Table 2:** Minimum inhibitory concentration (MIC) of methanolic extracts of R. californica and U. californica (mg/ml)

Plants	*B. cereus* ATCC® 14579	*S. aureus* ATCC® 25923	MRSA ATCC® BAA-1683	*M. smegmatis* ATCC® 10143
*R. californica* Leaves	4.0 ± 0	6.0 ± 0*	6.0 ± 0*	5.0 ± 1.4*
*U. californica* Leaves	4.0 ± 0	4.3 ± 0.5	5.5 ± 1.0*	4.7 ± 1.2
*U. californica* Bark	3.3 ± 1.2	4.0 ± 0	5.0 ± 1.4*	6.0 ± 2.1
Methanol	--	--	--	--

Values represent averages ± SD. --, not active; most experiments were repeated three times (*n* = 3); *n = 2

### Chemical profiling

Alkaloids, flavonoids, cardenolides and saponins were detected in the methanolic extracts of *R. californica* leaves and *U. californica* leaves and bark. Leucoanthocyanidins were detected in both *U. californica* leaves and bark but not in *R. californica* leaves. Tannins were detected in the leaves of both *R.* and *U. californica* but not in the bark of *U. californica*. Steroids were only detected in the bark of *U. californica* but not in the leaves of *U.* or *R. californica.* Finally, quinones were detected only in the leaves of *R. Californica* (Table 3)*.*

**Table 3:** Extract yield and chemical profile of methanolic extracts of R. californica and U. californica

	Plants		
	*R. californica*	*U. californica*	
	Leaves	Leaves	Bark
Extract Yield (% w/w)	13.9	28.9	5.75
Alkaloids	+	+	+
Flavonoids	+	+	+
Leucoanthocyanidins	-	+	+
Cardenolides	+	+	+
Tannins	+	+	-
Saponins	+	+	+
Steroids	-	-	+
Quinones	+	-	-

+, present; −, absent

## Discussion

Despite the alarming rise in AMR over the last several years, resistance to antimicrobial drugs is nothing new. In 1945— two years after the mass-production of penicillin— over 20 % of *S. aureus* strains isolated from hospitalized patients were penicillin-resistant [22]. Subsequent antibiotics such as streptomycin, tetracycline, methicillin, cephalothin, gentamicin, cefotaxime, and linezolid all followed suit, resulting in the emergence of resistant bacterial strains one to four years after their introduction [22]. History indicates that for every new antimicrobial drug developed, a proportion of bacteria will become resistant to it and pose a greater threat to public health. Therefore, alternative strategies must be considered if antimicrobial resistance is to be successfully combated. One alternative is the use of medicinal plants, which for many cultures continue to play a crucial role in the primary care of patients [3]. In fact, the World Health Organization supports combining western with traditional medicinal practices in order to develop truly effective remedies for our modern health challenges [23].

It is widely accepted that the antimicrobial activities and physiological effects from plant therapies are due to the biosynthesis of secondary metabolites produced by plants as a defense mechanism [24]. Very limited chemical profiling work has been done on *R. californica* (as mentioned previously, earlier chemical studies reported the presence of anthraquinones [10, 11] in the genus *Rhamnus*), while terpenoids [9, 12], alkaloids [13], and flavonoids [14] are reported as major constituents of *U. californica* [15] with cyanogens, sugars and tannins as minor constituents [12]. Our study is the first to detect the presence of quinones, alkaloids, flavonoids, cardenolides, tannins and saponins in *R. californica* (leaves). In addition, alkaloids, flavonoids, cardenolides, saponins, tannins (leaves), and steroids (bark) were detected in *U. californica*. Individually, each of these classes of compounds varies considerably in antimicrobial capacity [25]. At the same time, some of the structural classes found in *R.* and *U. californica* have recently shown promising anti-MRSA potential. Specifically, a structural analog of the anthraquinone emodin, found in the genus *Rhamnus*, and a novel class of synthetic antibacterial cationic anthraquinones have shown potent antibacterial effects against MRSA [26, 27]. Likewise, structural analogs of flavonoids kaempferol, quercetin and eriodictyol [28], found in *U. californica*, and plant extracts containing terpenoids umbellulone, 1,8-cineole, α-terpineol and thymol among others [29–31] have shown anti-MRSA activity. Interestingly, even though 1,8-cineole exhibits little inherent antimicrobial activity, it has been shown to enhance the potency of other antimicrobials against MRSA [31]. Therefore, it is likely that the antimicrobial activity observed herein is due to the synergistic effects of all or most of the secondary metabolites present in each extract [31, 32]. In addition, the many compounds in the extracts may affect the bacteria via several different mechanisms, thus decreasing the likelihood of selecting for a resistant strain.

Our study is the first to report the antimicrobial potential of the methanolic extracts of leaves and bark of *R. californica* and *U. californica*, all of which significantly inhibited growth of Gram-positive bacteria such as *B. cereus*, *S. pyogenes*, and *S. aureus*, as well as the acid-fast organism *M. smegmatis*. Most intriguing was the discovery that these plant extracts were effective at controlling the growth of MRSA, one of the most ominous AMR strains, with MICs of 3.3-6.0 mg/ml. While we acknowledge that these MIC values are relatively high (in comparison to the generally accepted notion that plant extracts have significant antimicrobial activity if their MIC values are 100 μg/ml or lower), the fact that the two Gram-negative organisms *E. coli* and *P. aeruginosa* were virtually unaffected by these extracts is noteworthy and indicates that the antimicrobial effects on the Gram-positive organisms are specific and thus significant. This complete lack of activity on the Gram-negative organisms is most likely due to the protective nature of the outer membrane of their cell walls. Furthermore, MIC values similar to those obtained in this study have been reported recently by other researchers, such as: Farooqui et al., who tested the antibacterial activity of *C. Sinensis* and *J. Regia* against multidrug-resistant bacteria including MRSA [33]; Siwe Noundou et al., who demonstrated the antibacterial activity of *A. floribunda* against *B. cereus* and *S. aureus* [34]; and Zuo et al., who tested the antibacterial activity of 19 Chinese medicinal plants against MRSA [35]. Interestingly, the study by Zuo et al. showed that the MIC value of the isolated active component was 20-times greater than that of the crude extract [35].

Due to the limited amount of starting material available it was not possible to test the antimicrobial activity of our plant extracts on an extensive number of microorganisms. However, we successfully assessed the *in vitro* antimicrobial activity of these plant extracts against a MRSA strain (ATCC® BAA-1683, originally isolated from a patient at the Henry Ford Hospital in 2004) and a variety of Gram-positive and Gram-negative bacteria. Further investigation of the antimicrobial activity of these extracts against other clinical isolates of MRSA is warranted.

## Conclusion

Our results demonstrate the antimicrobial properties of *R.* and *U. californica* and illustrate their promising anti-MRSA potential. The findings reported herein give scientific credence to the traditional medicinal uses of these plants by the indigenous peoples of California and suggest that extracts of *R.* and *U. californica* merit further chemical study as natural antibiotics to identify the secondary metabolite(s) responsible for their antimicrobial activity, since such structures could serve as valuable therapeutic anti-MRSA leads.
